# Ethical and legal considerations of artificial intelligence applications in psychiatric violence risk assessment: A scoping review protocol

**DOI:** 10.1371/journal.pone.0334649

**Published:** 2025-10-21

**Authors:** Andrew Lee, Daniel Buchman, Katrina Hui, Sophie Nunnelley, Stephanie Penney, Terri Rodak, Juveria Zaheer, Laura Sikstrom

**Affiliations:** 1 Temerty Medicine Department of Psychiatry, University of Toronto, Toronto, Ontario, Canada; 2 Centre for Addiction and Mental Health, Toronto, Ontario, Canada; 3 Dalla Lana School of Public Health, University of Toronto, Toronto, Ontario, Canada; 4 Joint Centre for Bioethics, University of Toronto, Toronto, Ontario, Canada; 5 St. Michael’s Hospital, Toronto, Ontario, Canada; 6 Lincoln Alexander School of Law, Toronto Metropolitan University, Toronto, Ontario, Canada; 7 Department of Anthropology, University of Toronto, Toronto, Ontario, Canada; PLOS: Public Library of Science, UNITED KINGDOM OF GREAT BRITAIN AND NORTHERN IRELAND

## Abstract

Violence risk assessment is a critical component of psychiatric practice, with significant clinical, ethical, and legal implications. Psychiatric patients at high risk of violence often face interventions including restraints, intramuscular injections, and involuntary hospitalization. Agitated and aggressive behaviours from patients have been linked to high hospital costs due to increased length of stay, readmissions, increased medication use, staff injury, and need for high acuity monitoring. Traditional risk assessment tools can be time intensive and have poor generalizability to civil populations. Recent advances in artificial intelligence (AI) have the potential for enhancing the precision of violence risk assessments. Although AI can address the technical issues of risk assessment, its implementation will raise new ethical and legal challenges. In psychiatry, AI-assisted violence risk assessment intersects with mental health law, particularly criteria for preventive detention and the ethical boundaries of AI-driven decisions. There have been some early concerns about racial bias, lack of transparency, accountability, and disruption to current practices in psychiatric care. To our knowledge, there have been no efforts to synthesize the ethical and legal implications for this particular use case. To address these gaps, we conducted a scoping review to map the literature on the ethical and legal considerations of AI in violence risk assessment in acute psychiatry.

## Introduction

### Violence in psychiatry – Prevalence, costs and implications

Violence risk assessments are an important aspect of comprehensive psychiatric assessments, as violence has a significant impact on patients, healthcare providers, institutions, and society more broadly. Violence and aggression are conceptualized on a spectrum of severity of harm. Aggression is defined as behaviour directed to another individual with the intent to cause harm, which can take the form of physical or verbal acts. Violence is considered an extreme form of aggression that has severe harm as its goal [1]. Agitated and aggressive behaviours from patients have also been linked to symptom burden, more complicated recovery trajectories and increased economic costs, including prolonged hospital stays, increased rates of readmission, higher use of medications, loss of productivity following staff injury, and the need for higher acuity care [2]. Additionally, violent incidents committed in hospital settings can further erode public confidence in the system and contribute to more restrictive and stigmatizing practices [3]. A recent review suggests that up to 1 in 5 individuals who are admitted to inpatient psychiatry may become physically violent [4]. Due to the prevalence and costs of violence in the psychiatric setting, it is crucial for clinicians to consider the risk of violence in their assessments.

Where a patient is estimated to be at high risk for violence there can be varying implications along a continuum of verbal de-escalation to involuntary hospitalization and mandatory treatment [5,6]. The history of “civil commitment” has long included concern for the *dangerousness* or *risk of harm* to others as a qualifying criterion [7]. The terminology of “dangerousness” and “risk of harm” is used – often interchangeably –in mental health laws around the world [8]. Although dangerousness has been widely accepted as a criterion for civil commitment, its use is not universal. Countries such as Italy, Spain, and Sweden have omitted the dangerousness criterion in favour of treatment-oriented criteria, such as the presence of a serious mental disorder, need for treatment, and the inability to provide informed consent for care. Most other European Union countries include some concept of “danger” or risk to others in their criteria [9]. Because of these laws, psychiatric risk assessments can have important implications for patients’ rights and freedoms.

The ethics of preventive detention for the reason of risk of harm to others remains controversial. There are concerns that preventative detention can lead to a disproportionate infringement of patients’ rights where the perceived risk may never materialize. Additionally, the bias that assumes those with mental illness are dangerous may lead to discrimination, where they are disproportionately controlled to be treated and treated to be controlled [10]. Such assumptions conflict with the evidence; patients with mental health are more likely to be victims of violence than perpetrators [11,12]. Ultimately, there are concerns about how the concept of dangerousness is operationalized and used to detain patients. These concerns are compounded by factors such as clinicians concerns about medicolegal liability, as well as doubts surrounding the reliability and validity of violence risk assessments. Furthermore, there is some evidence of racial and socioeconomic biases that lead to disproportionate use of restraints for behavioural health related visits to the emergency department after patients are detained [13]. Thus, the ethical and legal concerns regarding violence risk extend beyond accuracy of our assessment tools, to the actions taken when individuals are identified as high risk.

### Violence risk assessment tools – Reliability and validity

Significant advances have been made in identifying risk factors for violence, and these advances have been translated into validated tools for assessing violence risk (e.g., the Dynamic Appraisal of Situational Aggression [DASA; [14]]; the Historical, Clinical, and Risk Management-20; [HCR-20; [15]]). Risk factors for violence that have received consistent empirical support include static, or relatively stable historical variables (e.g., man gender, young age at first offense, history of substance use or relationship problems), as well as dynamic, or theoretically modifiable indicators (e.g., active psychiatric symptoms, current substance use, insight, mood and affect, treatment or supervision compliance) [4]. Assessment tools often include some combination of these factors but vary in terms of their focus on static versus dynamic risk, the type of violence for which risk is estimated (e.g., inpatient aggression, intimate partner or sexual violence), as well as the time period over which the risk estimate is presumed to be valid (e.g., imminent versus long-term risk forecasts).

Research on the reliability and validity of violence risk assessment tools and practices has been largely conducted in offender and forensic mental health samples, while the applicability to civil populations is less emphasized [16]. There has also been growing recognition of the limits of predictive accuracy achieved by most published risk assessment instruments, with the observation that correlations between risk estimates and violence are modest and rarely exceed 0.40 [17,18]. Furthermore, research on the predictive accuracy of risk assessment instruments continues to outpace research on the utility of these tools for risk management and violence prevention, such that questions remain as to whether these tools assist in reducing risk and averting harmful outcomes [19,20].

### Artificial intelligence in violence risk assessment – Clinical, ethical and legal considerations

Given the observed limitations associated with existing tools used to conduct violence risk assessments, there has been growing interest in using artificial intelligence (AI) applications to potentially enhance the precision, accuracy, and clinical utility of these assessments in predicting violence [21]. AI and more specifically machine learning (ML) are technologies that can conduct complex analyses of large datasets (e.g., data from the brain, behaviour, and genes) that potentially allow for more accurate prediction of future events, while iteratively improving over time on their own [22]. Perhaps one of the greatest advantages in using ML applications to assist with violence risk assessment is conferred through its ability to synthesize and analyze very large amounts of complex data. Technical improvements in the volume of information that can be efficiently reviewed, particularly for individuals who have amassed large amounts of information in their health record, may substantially improve our ability to estimate the probability of violence and on varying time horizons.

Within the current climate of rapidly advancing ML applications and systems, the clinical practice of violence risk assessment can provide a unique case study for how ML and psychiatry will need to contend with both ethical and legal constraints which have a direct effect on how these technologies are regulated, evaluated, and potentially used or misused. ML models are currently being developed and tested with respect to violence risk predictions in psychiatric populations with varying levels of validity and reliability [23–25]. There is currently a breadth of models being created which incorporate varying numbers and types of predictive variables (e.g., demographic data, imaging data, violence risk scores) and employ different algorithmic approaches (e.g., decision trees, random forests, naive Bayes, gradient boosting machines, support vector machines, neural networks, and some variations and combinations of techniques) [25]. Pilots of AI-driven risk assessment tools and “early warning systems” have been seen at institutions such as Duke University Health System hospitals and Waypoint Centre for Mental Health Care [26,27].

From an ethical perspective, it may be difficult to ascribe accountability for issues that arise with the use of these technologies [28]. A particularly pernicious issue concerns the potential for embedded bias based on race or ethnicity in ML generated risk predictions. Available evidence suggests that risk instrument validity diminishes as samples approach greater ethnic heterogeneity [29,30] and there is insufficient evidence at present to equivocally show that commonly used risk assessment instruments demonstrate adequate cross-cultural validity or equivalence. Using ML to supplement risk decisions may serve to amplify some of these issues [31]. Notably, the clinical practice of assessing risk in civil psychiatric patients (versus forensic or offender populations, for example) also carries with it different legal thresholds and implications, as the restriction of rights and freedoms are governed by civil versus criminal law. Many AI tools are being examined in forensic populations, which may have similar limitations when applied to civil populations as existing risk assessment tools. For these ML tools to be successfully implemented in clinical practice, it is essential to consider the intersecting clinical, ethical, and legal questions.

### Rationale for review

To our knowledge, there have been no efforts to map the literature on the ethical and legal issues raised by AI-enabled violence risk assessments. There appear to be competing positions in the literature. Some suggest AI-use does not introduce serious or unique challenges beyond those found in other forms of violence risk assessment. Others highlight concerns, for instance, that AI-use could entrench bias and exacerbate disparities. The range of perspectives requires synthesis and analysis [32,33]. Beyond the specific ethical and legal issues raised in this context, violence risk assessment is also a useful case study for understanding how ML may disrupt existing clinical practices and present intersecting ethical and legal challenges more broadly.

Addressing these gaps, this scoping review will synthesize the current literature discussing legal and ethical issues with AI-based violence risk assessment, identifying the key concepts, and their conceptual boundaries [34]. By identifying the pertinent ethical and legal considerations this review will provide an important knowledge base. We hope to utilize the knowledge from this review to inform future ethical guidelines for use of AI to enhance risk assessment in mental health contexts.

## Methods

### Scoping review protocol design

Our review is guided by the scoping review framework described by Arksey and O’Malley [34]. As compared to a systematic review, a scoping review offers a rigorous and transparent method for mapping an area of research, providing flexibility to address a range of topics and study designs. The scoping review methodology also supports a narrative and descriptive review of the field of research. A limitation is the absence of any appraisal of the quality of included studies. A scoping review is most conducive to an interdisciplinary and heterogenous subject such as this one. Our research question is broad to fully encompass the range of discussions of ethical and legal issues, which fits well with the methodology of a scoping review. Our main goal is to describe the literature on the ethical and legal issues raised by the use of AI for violence risk assessment in psychiatric settings. The objective is to generate insights and guidance that will be useful for clinicians, policy makers, legal experts, researchers, and ultimately patients.

Based on the methodology outlined by Arksey and O’Malley, we will adhere to the following phases: 1) identifying the research question 2) identifying relevant records; 3) selecting relevant records; 4) charting the data 5) collating, summarizing, and reporting the results and 6) consulting with the interdisciplinary research team [34]. We will also outline the gaps in the literature and recommend for future research directions.

### Planning and research question

Our research question is: What are the ethical and legal considerations for the implementation of machine learning in violence risk assessment for psychiatric populations? This question was developed by an interdisciplinary group of researchers including clinicians (psychiatry, clinical psychology, nursing), bioethicists, data scientists, medical anthropologists, and persons with lived experience. An iterative approach was taken to shape a research question that will be clinically, ethically, and legally relevant and applicable.

### Search strategy and identifying relevant studies

In collaboration with the research team, a health sciences librarian will develop a core search strategy in MEDLINE (Ovid) based on the research question with iterative changes informed by preliminary searches, metadata from gold standard articles, and finalized eligibility criteria. The strategy will then be translated for use in the other selected databases: Scopus (Elsevier), Embase (Ovid), APA PsycINFO (Ovid), Cumulative Index to Nursing and Allied Health Literature – CINAHL (EBSCO), International Bibliography of the Social Sciences – IBSS (ProQuest), Applied Social Sciences Index & Abstracts – ASSIA (ProQuest), Philosopher’s Index (ProQuest), IEEE Xplore, and Law Journal Library (Hein). All strategies will use database-specific subject headings, keywords in natural language, and advanced search operators to capture the relationship between four concepts: artificial intelligence, violence risk, law and ethics. Our “ethics” concept will be intentionally broad and will incorporate terminology from philosophical, socio-legal, and social justice frameworks, such as “moral*”, “informed consent”, and “prejudic*”, as well as terms to capture articles likely to touch on issues of equity (e.g., “vulnerable”, “power*”, “co-creat*”). Publication date will be limited to January 1, 2014 to the July 30 2025, as this is a contemporary topic that has evolved in the past 10 years. No study design or language limits will be applied. The full MEDLINE search strategy can be seen in S1 Appendix.

Reference lists of eligible articles will be hand searched for additional sources. A search for grey literature will also be conducted to identify any unpublished works and institutional reports through Google Scholar and a targeted search of selected websites. The study has been registered with the Open Science Framework (OSF) and can be found here: https://osf.io/qx2jy.

### Study selection and screening

We will complete two phases of screening based on predetermined criteria. Search results from the scholarly databases will be uploaded to Covidence [35], a web-based software platform that streamlines the production of systematic and other literature reviews. In the first phase of screening, two authors will independently screen titles and abstracts based on our eligibility criteria. Any discrepancies will be resolved by consensus, and if necessary, a third author will make the final determination. Two authors will then complete full-text reviews to identify articles meeting the inclusion and exclusion criteria. We will follow the Preferred Reporting Items for Scoping Review (PRISMA-ScR) checklist to guide our screening process (see S2 Appendix). We will include a diagram of our screening results through the four-phases outlined in the Preferred Reporting Items for Systematic Reviews and Meta-Analyses diagram [36].

### Data charting

Our data charting methods will be informed by the suggestions of Pollock and colleagues (31). We have outlined themes a priori for data extraction rather than using deductive-inductive coding to apply existing ethical and legal frameworks to our current research question [37]. The predetermination of themes will allow us to better map specific constructs, while identifying underrepresented considerations in the literature. We will extract data using a template in Covidence and chart definitions, statements, or arguments related to the pre-established concepts. We will extract data based on article characteristics (e.g., citation, year, country, study design, population, and purpose/objective), ethical and legal themes, and future recommendations.

Ethical and legal themes will be defined by existing frameworks and will undergo iterative changes in discussion with the interdisciplinary research team. Researchers will also chart any contextual representations of the established concepts, to ensure a broader capture of these themes even if specific terminology is not utilized. Subsequently, each of the two researchers will independently chart the included studies and then verify the accuracy of the data charting.

### Data analysis

The data analysis will include descriptive statistics of the article characteristics and qualitative thematic analysis. Data will be extracted based on the established framework and concepts and reviewed by researchers to determine the appropriateness of the framework to provide a map of the available evidence. The results will be summarized on the established framework. We will then consider the findings in the context of our research question and broader implications for practice and policy.

### Summarization and reporting of results

We will summarize the results of our scoping review both quantitatively and qualitatively. Quantitative data, such as descriptive statistics, will largely be described through the PRISMA diagram and basic tabulations of article characteristics (e.g., country of study). We will conduct basic qualitative coding of results from ethical and legal discussions under broader categories to delineate important concepts and definitions. These broader categories will be diagrammatically presented to display basic relationships within and between ethical and legal concepts as defined by best practices. Key findings will be organized thematically. Results will be presented using visual forms such as tables, charts, diagrams, and narrative forms, as appropriate.

### Study status and timeline

At the time of manuscript submission the study selection is ongoing. We anticipate that the data charting will be complete by November 2025, and data analysis and synthesis by December 2025. We anticipate that results will be available by March 2026.

### Dissemination and ethics

We plan to present this and associated works at ethics and psychiatry conferences nationally and internationally. We will publish the results of our scoping review in a peer-reviewed academic journal. The results will inform a qualitative study on the perceived implications of ML applications in violence risk assessment from lawyers, psychiatrists, and civilians involved in the consent and capacity board process, which is a tribunal that makes decisions regarding involuntary hospitalizations, community treatment orders, and incapacity to consent to treatment. Additionally, we will share the results of our study within our own organization (Centre for Addiction and Mental Health) to inform internal AI governance policies and procedures for implementing violence risk tools in clinical practice.

## Conclusion

Violence risk in the psychiatric population is of clinical, ethical, and legal importance. Innovations in risk prediction have been an important area of inquiry due to the social and economic costs associated with violence. Although AI in medicine is an evolving landscape of ethical and legal discussion, there has been limited focus on ML applications for risk prediction in psychiatry. It is imperative that we consider the potential implications of these technologies as violence risk is a complex phenomenon that can restrict a patients’ liberties, dictate treatment plans, and determine the level of coercion they are subject to. Our scoping review will map the current landscape of ethical and legal considerations in ML applications of violence risk assessment and inform future research directions.

## Supporting information

S1 Appendix Sample search. (DOCX)

S2 Appendix PRISMA-ScR-checklist. (DOCX)
