# Supplementary material for: Ethical and legal considerations of artificial intelligence applications in psychiatric violence risk assessment: A scoping review protocol
Source: PLoS One. 2025 Oct 21;20(10):e0334649. doi: 10.1371/journal.pone.0334649 (PMC12539692; doi:10.1371/journal.pone.0334649)
Supplement: S1 Appendix — (DOCX) [file pone.0334649.s001.docx]

| **Ovid MEDLINE: Epub Ahead of Print, In-Process & Other Non-Indexed Citations, Ovid MEDLINE® Daily and Ovid MEDLINE® <1946-Present>** | | |
| --- | --- | --- |
| 1 | artificial intelligence/ or computer heuristics/ or expert systems/ or fuzzy logic/ or knowledge bases/ or machine learning/ or natural language processing/ or neural networks, computer/ or robotics/ or sentiment analysis/ | 180658 |
| 2 | machine learning/ or deep learning/ or supervised machine learning/ or support vector machine/ or unsupervised machine learning/ | 79911 |
| 3 | neural networks, computer/ or deep learning/ | 74464 |
| 4 | medical informatics/ or medical informatics applications/ or medical informatics computing/ | 16245 |
| 5 | Data Mining/ | 11456 |
| 6 | artificial* intelligen*.mp. | 86073 |
| 7 | (AI not american indian*).mp. | 64449 |
| 8 | machine learning.mp. | 138645 |
| 9 | deep learning.mp. | 76487 |
| 10 | (natural language process* or NLP*).mp. | 17092 |
| 11 | neural network*.mp. | 133866 |
| 12 | (support vector* or SVM* or SVN* or SVR*).mp. | 53021 |
| 13 | (data mining or datamining).mp. | 23258 |
| 14 | big data*.mp. | 17197 |
| 15 | (predictive adj3 analy*).mp. | 12173 |
| 16 | or/1-15 [AI] | 490158 |
| 17 | exp Risk Assessment/ | 324465 |
| 18 | risk*.ti,kf,hw. | 1732599 |
| 19 | (risk* adj5 (assess* or predict* or identif* or detect* or manag* or factor* or audit* or estimat*)).mp. | 2036887 |
| 20 | needs assessment*.mp. | 39693 |
| 21 | compas.mp. | 143 |
| 22 | or/17-21 [risk assess] | 2367154 |
| 23 | violence/ or adverse childhood experiences/ or domestic violence/ or gender-based violence/ or gun violence/ or intimate partner violence/ or physical abuse/ or rape/ or terrorism/ or torture/ or workplace violence/ | 70745 |
| 24 | exp Homicide/ | 24950 |
| 25 | exp Sex Offenses/ | 28916 |
| 26 | forensic psychiatry/ or "commitment of mentally ill"/ or insanity defense/ | 16142 |
| 27 | (forensic* adj3 (psychiatr* or psycholog* or nurs* or mental* or system* or setting* or facility or facilities or institut* or environment* or ward or wards or unit or units or patient* or inpatient* or outpatient*)).mp. | 16680 |
| 28 | violen*.mp. | 99189 |
| 29 | assault*.mp. | 19173 |
| 30 | aggressi*.mp. | 287050 |
| 31 | agitat*.mp. | 28461 |
| 32 | (property adj3 (damag* or destr*)).mp. | 1259 |
| 33 | ((crisis or crises) adj3 (behav* or manag*)).mp. | 4222 |
| 34 | ((physical* or sex* or domestic* or spous* or partner*) adj3 abus*).mp. | 38087 |
| 35 | or/23-34 [violence] | 491823 |
| 36 | 16 and 22 and 35 | 719 |
| 37 | ((risk* or assess* or predict* or identif* or detect* or manag* or factor* or audit* or estimat*) adj3 (violen* or intimate partner violence or IPV or domestic violence or sexual* violen* or gender based violence or homicid* or femicid* or assault* or sex* offen* or aggressi*)).mp. | 36430 |
| 38 | ((risk* or assess* or predict* or identif* or detect* or manag* or factor* or audit* or estimat*) adj5 ((physical* or sex* or domestic* or spous* or partner*) adj3 abus*)).mp. | 6297 |
| 39 | 16 and (37 or 38) | 512 |
| 40 | exp ethics/ | 159198 |
| 41 | exp morals/ | 187377 |
| 42 | exp patient rights/ or exp human rights/ or civil rights/ | 160228 |
| 43 | exp confidentiality/ | 57855 |
| 44 | jurisprudence/ | 30039 |
| 45 | social values/ | 20251 |
| 46 | (ethic* or unethic*).mp. | 273960 |
| 47 | bioethic*.mp. | 25457 |
| 48 | es.fs. | 79011 |
| 49 | (human right* or patient* right* or "civil right*" or "bill* of right*").mp. | 47776 |
| 50 | bias*.mp. | 354294 |
| 51 | (equit* or inequit*).mp. | 80451 |
| 52 | virtue*.mp. | 23224 |
| 53 | moral*.mp. | 59140 |
| 54 | principle*.mp. | 349303 |
| 55 | autonom*.mp. | 224439 |
| 56 | (value* adj3 (theor* or philosoph* or human* or person* or inherent* or instrumental* or intrinsic* or extrinsic* or social*)).mp. | 49214 |
| 57 | humanis*.mp. | 10094 |
| 58 | informed consent.mp. | 76740 |
| 59 | conflict* of interest.mp. | 18302 |
| 60 | privacy.mp. | 32716 |
| 61 | confidential*.mp. | 40782 |
| 62 | surveillance.mp. | 314733 |
| 63 | criminaliz*.mp. | 1550 |
| 64 | (beneficen* or non-malefic* or nonmalefic*).mp. | 5904 |
| 65 | accountab*.mp. | 29276 |
| 66 | fair*.mp. | 111564 |
| 67 | ((person* or patient*) adj3 (centre* or center*)).mp. | 108743 |
| 68 | therapeutic alliance.mp. | 3924 |
| 69 | (justice or injustice*).mp. | 40574 |
| 70 | humility.mp. | 2223 |
| 71 | epistemolog*.mp. | 6609 |
| 72 | transparen*.mp. | 77353 |
| 73 | social licen*.mp. | 165 |
| 74 | neuroethic*.mp. | 836 |
| 75 | care.mp. | 2785416 |
| 76 | wisdom.mp. | 9954 |
| 77 | courage.mp. | 2847 |
| 78 | compassion*.mp. | 18273 |
| 79 | equipoise.mp. | 2276 |
| 80 | discriminat*.mp. | 344383 |
| 81 | prejudic*.mp. | 32490 |
| 82 | marginaliz*.mp. | 13075 |
| 83 | disadvantag*.mp. | 105855 |
| 84 | vulnerab*.mp. | 221309 |
| 85 | ethnic*.mp. | 242515 |
| 86 | (race or racial* or racism or racist).mp. | 222997 |
| 87 | gender*.mp. | 488662 |
| 88 | (sexis* or homophob* or transphob* or ageis* or ableis* or classis*).mp. | 10870 |
| 89 | (public* adj2 interest*).mp. | 5605 |
| 90 | (oppress* or antioppress*).mp. | 4509 |
| 91 | power*.mp. | 698506 |
| 92 | actuar*.mp. | 24783 |
| 93 | responsibility.mp. | 81340 |
| 94 | data sovereignty.mp. | 135 |
| 95 | (cocreat* or co-creat* or coprodu* or co-produc* or codesign* or co-design*).mp. | 16855 |
| 96 | ((patient* or client* or "user*" or family or families or lived experience) adj3 (engag* or consult* or collab* or lead or led)).mp. | 73703 |
| 97 | stigma*.mp. | 63384 |
| 98 | or/40-97 [ethics/equity] | 6251139 |
| 99 | (36 or 39) and 98 | 462 |
| 100 | limit 99 to yr="2014 -Current" | 405 |
